# *Cipactlichthys* *scutatus*, gen. nov., sp. nov. a New Halecomorph (Neopterygii, Holostei) from the Lower Cretaceous Tlayua Formation of Mexico

**DOI:** 10.1371/journal.pone.0073551

**Published:** 2013-09-04

**Authors:** Paulo M. Brito, Jesus Alvarado-Ortega

**Affiliations:** 1 Departamento de Zoologia, Instituto de Biologia, Universidade do Estado do Rio de Janeiro, Rio de Janeiro, Brazil; 2 Departamento de Paleontología, Instituto de Geología, Universidad Nacional Autónoma de México, Mexico City, Distrito Federal, Mexico; Institut de Biologia Evolutiva - Universitat Pompeu Fabra, Spain

## Abstract

Based on specimens from the Lower Cretaceous Tlayua Formation of Mexico, we describe a new genus and species of Halecomorphi, *Cipactlichthys* *scutatus* gen. et sp. nov, which exhibits several diagnostic characters such as the dermal bones and the scales with ganoin and highly ornamented by numerous tubercles and ridges; parietal slightly longer than wide with approximately the same length as the frontal; jaws extending far, below the posterior orbital margin, reaching the posterior third of the postorbital plate; maxilla with a convexly rounded posterior margin; pectoral fin margins slightly convex; first ray of pectoral fin very long, reaching the posterior edge of the pelvic fin; about 37 preural vertebrae and 7 Ural centra; a series of hypertrophied scales just posterior to the cleithrum; arrangement of flank scales with two rows of deep scales; a series of dorsal and ventral scutes forming the dorsal and ventral midline, between the dorsal and anal fins and the caudal fin. A phylogenetic analysis including two outgroups and eleven neopterygians confirmed the monophyly of the Holostei as well as the monophyly of the Halecomorphi, although this last clade is weakly supported. *Cipactlichthys* *scutatus* was hypothesised as the sister-group of the (Ionoscopiformes + Amiiformes).

## Introduction

Contrasting with the poor diversity of living halecomorphs represented today by only one taxon, *Amia* *calva* , restricted to the freshwaters of eastern North America, the fossil record of halecomorphs sensu Grande and Bemis [1] is highly diverse (e.g., Parasemionotiformes, Ophiopsidae, Ionoscopidae, Oshunidae, Caturidae, Sinamiidae, Amiidae, and some others).

Halecomorphs are neopterygians that were regarded since the 1970s as the sister group of the teleosts [2], although recently Grande [3] in a comprehensive study of the Ginglymodi, hypothesised the sister group relationship between this group and the Halecomorphi, resurrecting the Holostei.

The earliest halecomorphs occur in the Triassic although they are most diverse in the Late Jurassic and Early Cretaceous. However, new studies show that the neopterygian crown radiation happened in the Late Paleozoic [4], indicating a much earlier age for the holosteans as well as for the halecomorphs.

Among the Mesozoic fish faunas of Mexico, halecomorphs are represented by ophiopsids [5], ionoscopids [6], and amiids [1], all well represented in the Tlayua Formation.

The Tlayua Formation is dated as Early Cretaceous (Albian) based mainly on ammonites and belemnites [7–9]. This lithological unit is interpreted as an epicontinental marine deposit, within a basin under restricted and shallow conditions or an open marine basin, with weak continental inﬂuence [10–12]. The Tlayua Formation comprises a series of laminated limestones that split easily into thin slabs, for which it is worked as a paving stone. The exposed sequence exhibits some 30 to 34 m vertical thickness of mostly thinly bedded, yellow-reddish limestone strata of Early Cetaceous age (Albian) [11,13]. This locality is considered as a Konservat Lagerstätte [14] and is becoming increasingly known as one of the most important sources of Cretaceous fishes in Mexico.

Although about 80% of the Tlayuan fossil vertebrates are represented by well-preserved fishes [11,15], the assemblage also includes tetrapods and a considerable variety of marine invertebrates. The Tlayua Formation is considered nowadays as one of the most important fossiliferous localities of the western Tethys. It has a relationship not only with Tethyan fauna but also with coeval faunas found both in North and in South America.

**Figure 1:**
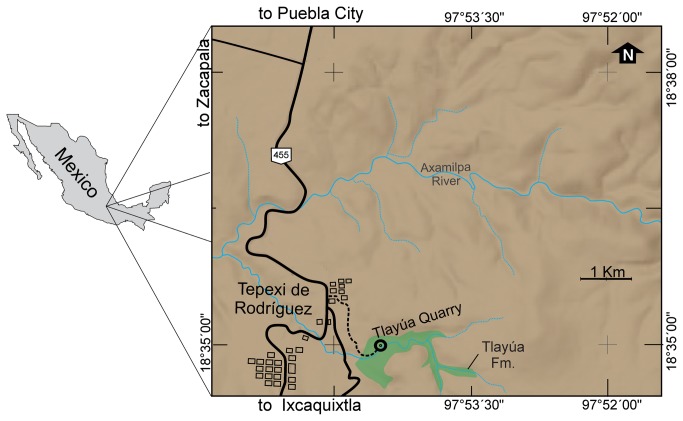
Map of the Tlayua Formation, State of Puebla, Mexico, indicating the location of the fossil localities of the Tlayua Formation. Cretaceous strata in green.

Here we describe a new genus and species of halecomorph holostean from the Cretaceous of Mexico and consider its affinities with regard to other halecomorph clades. The holotype was collected in the Tlayua Quarry, near the town of Tepexi de Rodriguez, Puebla (Figure 1). The fossils are permanently housed in the Colección Nacional de Paleontología of the Instituto de Geologia, Universidad Nacional Autónoma de México, Mexico, and registered under the acronym IGM.

## Methods

The new specimens described here (specimen numbers: IGM.6605 and IGM. 6606) were collected in the field by researchers of the Universidad Nacional Autonoma de Mexico (UNAM). After that they were curated in the official public collection of this institution that represents the National Paleontological Collection of Mexico. No specific permits were required for the described field-work.

Fossil and extant material used for comparison in this study are permanently housed in public collections in Brazil (Universidade do Estado do Rio de Janeiro and Departamento Nacional de Produção Mineral), and in France (Museum National d’Histoire Naturelle).

### Preparation

The two specimens described here come from the Middle Member of the Tlayua Formation [13], and are mainly preserved as mineralized skeletons in micritic limestone intercalated with argillaceous layers that show a characteristic red color due to hematite staining [15]. The holotype (IGM.6605) was prepared using the acid-transfer preparation technique [16]. The limestone was completely dissolved away using a 5 to 10% solution of formic acid buffered with calcium phosphate powder, leaving only the skeleton embedded in polyester resin. The second specimen (IGM.6606) was prepared using a pin vice and pneumatic air scribe.

Comparative specimens used in this study were also prepared using the techniques cited above except for *Watsonulus* *eugnathoides* , where the nodules had been cast in flexible molding silicone. The osteological data from extant species was obtained from material prepared using dermestid beetles.

### Terminology

We employed the traditional anatomical and directional terms used for describing actinopterygian osteology [1,17].

Institutional abbreviations: DGM Divisão de Geologia e Mineralogia, Departamento Nacional de Produção Mineral, Rio de Janeiro, Brazil; IGM Colección Nacional de Paleontología, Instituto de Geologia, Universidad Nacional Autónoma de México; UERJ Universidade do Estado do Rio de Janeiro, Rio de Janeiro, Brazil; MNHN Museum National d’Histoire Naturelle, Paris, France.

### Phylogenetic analysis

The phylogenetic analysis was based on a data matrix including 13 species of actinopterygian (ten fossil and three extant) as terminal taxa in order to test the phylogenetic position of these taxa among neopterygians. Comparative material, including that used in the phylogenetic analysis, is listed on Text S1. The characters that we used in this analysis are listed on Text S2. The sources of characters are provided in parentheses. Characters listed as “after” a speciﬁc published character indicates that our use of this character is different from the original usage (e.g. in deﬁnition of character states or differences in coding). These characters focus mainly on osteological data. Distribution of character states among taxa appear in the data matrix of Table S1. The search for most parsimonious trees and Bootstrap Analysis were performed using PAUP 4b10 [18].

### Nomenclatural Acts

The electronic edition of this article conforms to the requirements of the amended International Code of Zoological Nomenclature, and hence the new names contained herein are available under that Code from the electronic edition of this article. This published work and the nomenclatural acts it contains have been registered in ZooBank, the online registration system for the ICZN. The ZooBank LSIDs (Life Science Identifiers) can be resolved and the associated information viewed through any standard web browser by appending the LSID to the prefix "http://zoobank.org/". The LSID for this publication is: urn:lsid:zoobank.org:pub:E26DFFC6-189B-4590-8688-1995B010A78D. The electronic edition of this work was published in a journal with an ISSN, and has been archived and is available from the following digital repositories: PubMed, Central, LOCKSS.

## Results

### Systematic Palaeontology

Neopterygii Regan 1923 [19]

Holostei sensu Grande, 2010 [3]

Halecomorphi sensu Grande and Bemis, 1998 [1]

Order *Incertaesedis*

Family *Incertae* sedis

*Cipactlichthys* **gen. nov.**

urn:lsid:zoobank.org:act:606C1A1D-D46C-40D8-ACAB-F947FF8CF084

#### Derivation of name

*Cipactli*, named after the Aztec mythological sea monster (part fish and part reptile) plus *ichthys*, fish in Greek.

#### Diagnosis

As for the only known species below.

*Cipactlichthys* *scutatus* **sp. nov.**

urn:lsid:zoobank.org:act:DE53601A-A069-48E1-89E7-FAE47491FB44

#### Derivation of name

The specific epithet *scutatus*, in Latin, refers to the shield formed by the large dorsal and ventral scutes.

#### Holotype

**Figure 2:**
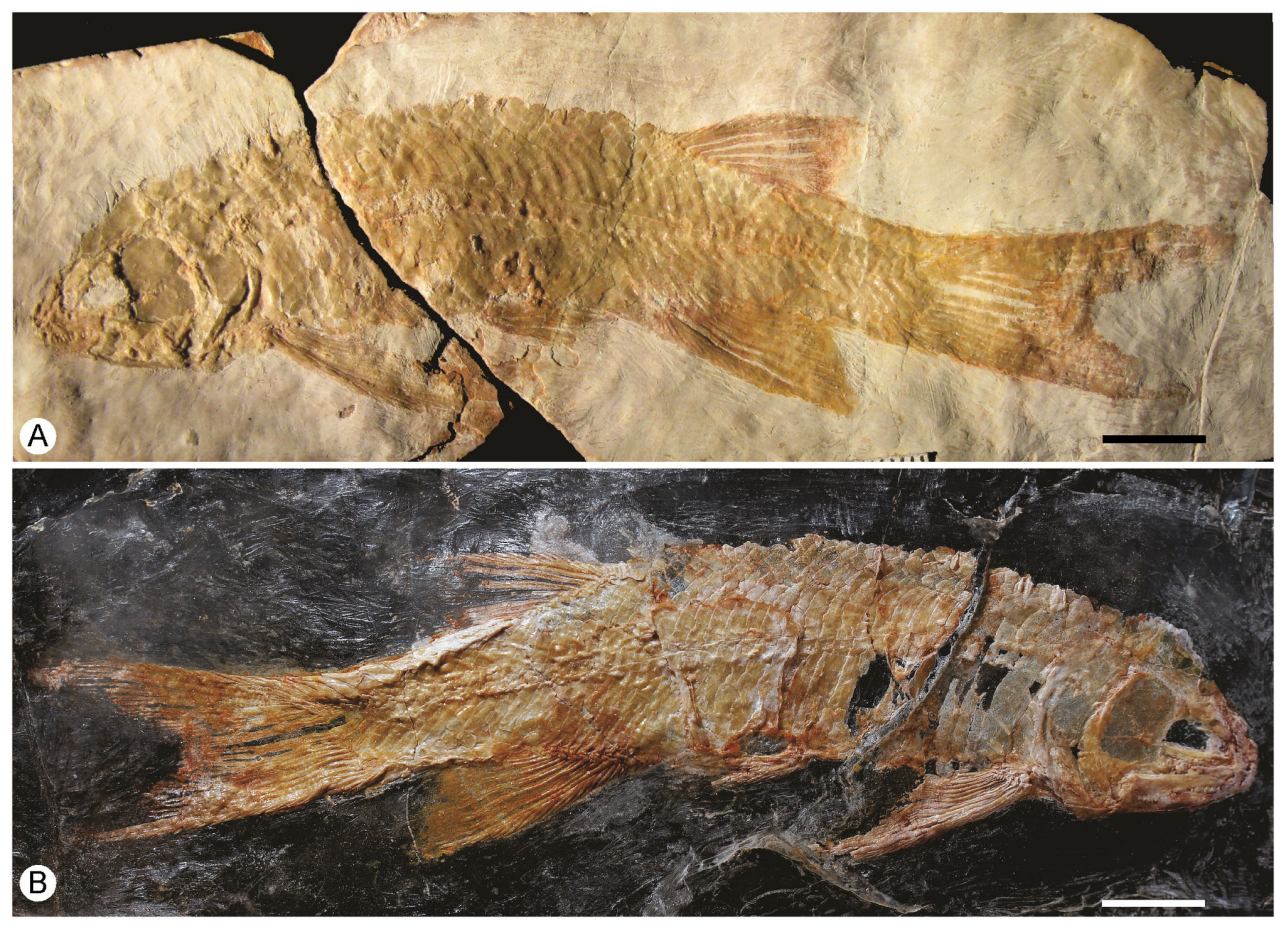
*Cipactlichthys* *scutatus* gen. et sp. nov. Holotype IGM.6605. A. Left side of a well-preserved complete skeleton before preparation. B. Right side of the same specimen after acid preparation. Scale bar equals 10 mm.

IGM.6605. An almost complete specimen, displaying its right side (Figure 2).

#### Referred specimen

**Figure 3:**
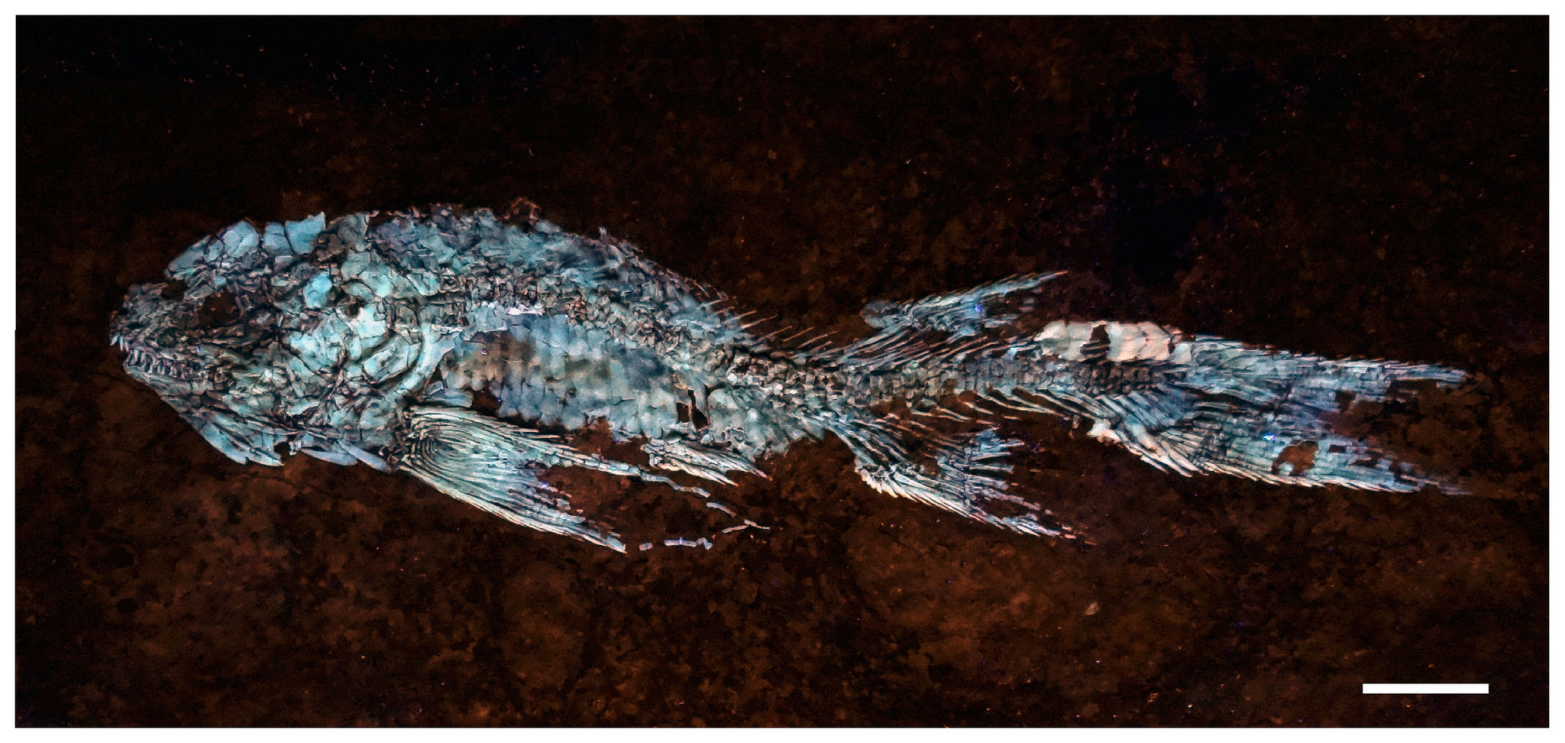
*Cipactlichthys* *scutatus* gen. et sp. nov. Specimen IGM.6606 (photograph under UV light). Scale bar equals 10 mm.

IGM.6606, an almost complete specimen, displaying its left side (Figure 3).

#### Horizon and type-locality

The specimens come from outcrops of the Tlayua Formation near the town of Tepexi de Rodríguez, State of Puebla, Mexico.

#### Diagnosis

Medium sized, elongate bodied halecomorph with dermal bones and scales covered with ganoin and highly ornamented by numerous tubercles and ridges; frontal almost equalin width throughout its length; parietal slightly longer than wide with approximately the same length as the frontal; extrascapular small and triangular; circumorbital series complete; posterior cheek region with three postorbital plates and narrow crescent-shaped preopercle; jaws extending far, below the posterior orbital margin, reaching the posterior third of the postorbital plate; maxilla with convexly rounded posterior margin; pectoral fin margins slightly convex; first ray of pectoral fin very long, reaching the posterior edge of the pelvic fin; caudal fin with forked outline and lobes of equal size; solid ossified smooth-sided vertebrae, deeper than long; about 37 preural vertebrae and 7 ural centra; a series of hypertrophied scales just posterior to the cleithrum; arrangement of flank scales with two rows of deep scales; a series of dorsal and ventral scutes forming the dorsal and ventral midline, between the dorsal and anal fins and the caudal fin.

### Description

This is a medium sized halecomorph of approximately 130 mm total length (TL) and about 100 mm standard length (SL). The deepest part of the body is constant between the pectoral and the anal fin. The head (HL), including the opercular series, measures approximately 22% of the standard length and 31% of the length from the snout to the origin of the dorsal fin. The dorsal fin is positioned in the posterior half of the body, and its origin is at about the same level as the anal fin. The pelvic ﬁn is located halfway between the pectoral and the anal ﬁns (Figures 2, 3).

The dermal bones and the scales are covered with ganoin and are highly ornamented on their external surfaces. The ornamentation is formed by numerous tubercles and ridges.

**Figure 4:**
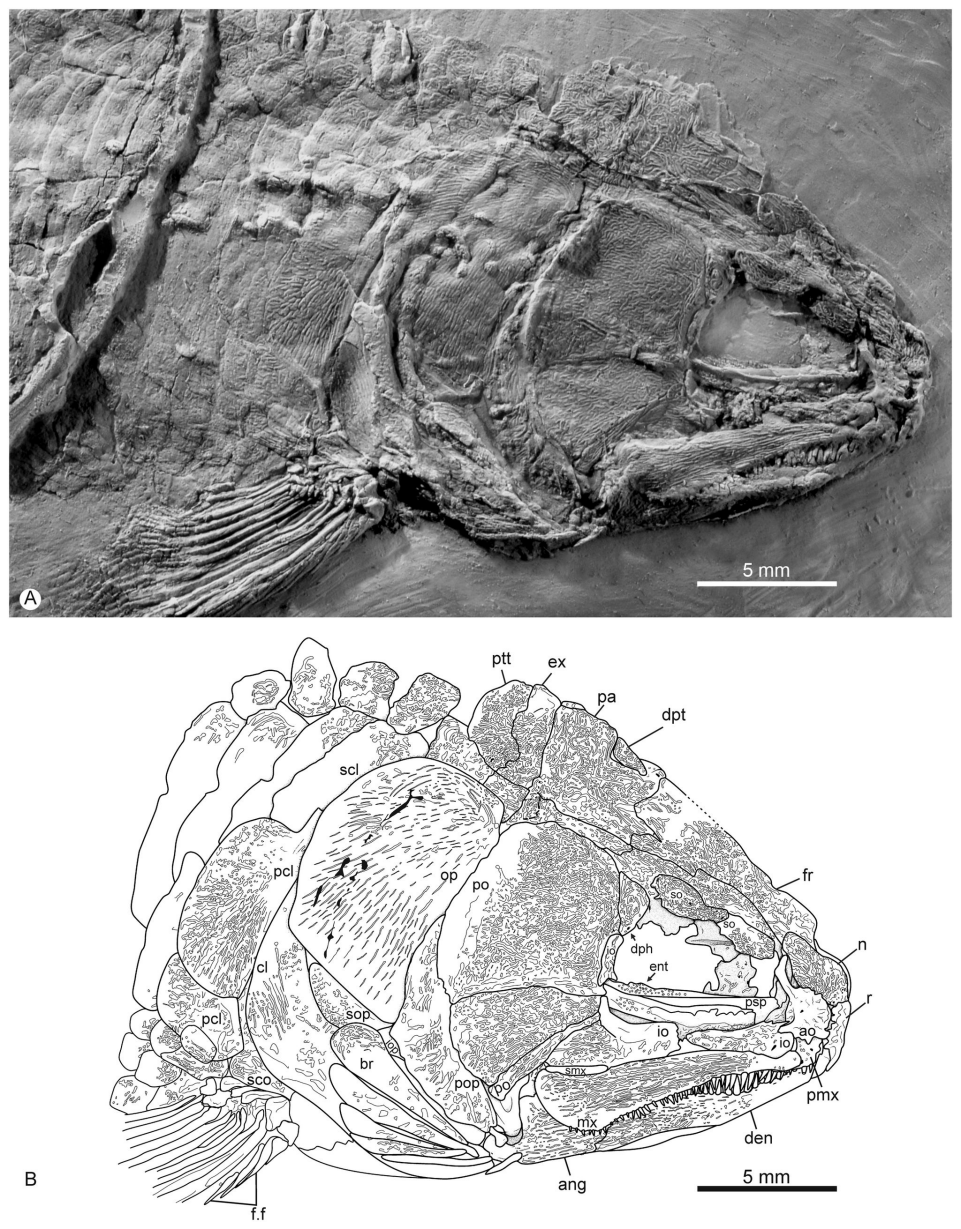
*Cipactlichthys* *scutatus* gen. et sp. nov. Holotype IGM.6605. A) photograph of the head region; B) anatomical interpretations. Abbreviations: ang, angular; ao, antorbital; br, branchiostegals; cl, cleithrum; den, dentary; dph, dermosphenotic; ent, endopterygoid; ex, extrascapular; f.f, fringing fulcra; fr, frontal; io, infraorbital; iop, interopercle; mx, maxilla; n, nasal; op, opercle; pa, parietal; pcl, postcleithrum; pmx, premaxilla; po, postorbital; pop, preopercle; psp, parasphenoid; ptt, posttemporal; ro, rostral; scl, supracleithrum; sco, scapulocoracoid; smx, supramaxilla; so, supraorbital; sop, subopercle. Scale bar equals 5 mm.

**Figure 5:**
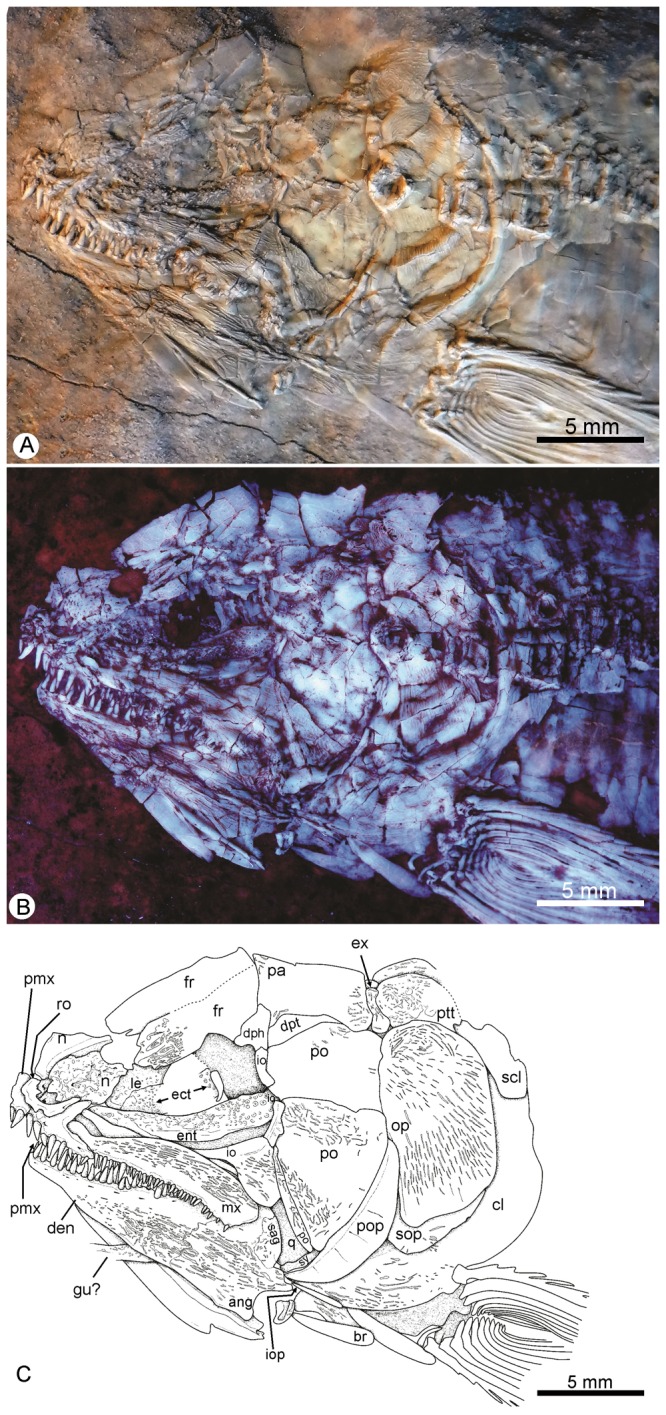
*Cipactlichthys* *scutatus* gen. et sp. nov. IGM.6606. A) photograph of the head region; B); photograph of the head region under UV light; C) anatomical interpretations. Abbreviations: ang, angular; br, branchiostegals; cl, cleithrum; den, dentary; dph, dermosphenotic; dpt, dermopterotic; ect, ectopterygoid; ent, endopterygoid; ex, extrascapular; fr, frontal; gu, gular plate; io, infraorbital; le, lateral ethmoid; mx, maxilla; n, nasal; op, opercle; pa, parietal; pmx, premaxilla; po, postorbital; pop, preopercle; psp, parasphenoid; ptt, posttemporal; q, quadrate; ro, rostral; sag, supraangular; scl, supracleithrum; sop, subopercle; sy, sympletic. Scale bar equals 5 mm.

The skull roof is preserved in lateral view on specimen IGM.6605 (Figure 4) and is partially preserved in dorsal view, where it is somewhat distorted, in IGM.6606 (Figure 5).

The rostral (Figures 4, 5) is a narrow tube-like and shallow element that overlies the anterior part of the premaxilla. The rostral houses the anteriormost commissure connecting the right and left antorbitals.

The antorbital is somewhat oval and strongly ornamented on its exterior surface. Its posterior margin borders the orbit closing anteriorly the infraorbital series. The bone presents three tubular canals; one antero-ventrally connecting to the rostral; one postero-dorsally contacting the nasal, and one postero-ventrally adjoining the lacrimal.

The nasal is longer than wide, with its anterior half slightly broader than the posterior one; both nasals seem to be in contact in the midline of the skull. The nasal overlaps the frontal posteriorly, and contacts the antorbital laterally.

The frontal lies just behind the nasal, and is the longest element of the dermal skull, having a length approximately three times that of the nasal, extending posteriorly to the parietal and dermopterotic. The bone is almost equalin width throughout its length. The suture between the frontals is almost straight, with very few interdigitations. The supraorbital sensory canal extends longitudinally, close to the lateral margin of the bone.

The parietal (Figures 4, 5) is subrectangular and well-developed, slightly longer than wide and having approximately the same length as the frontal. The suture with the frontal is sinuous. The lateral border of the parietal is almost linear and overlies the dermopterotic. The sensory canal reaches the anterior half of the bone. Pit-lines cannot be observed.

Lateral to the parietal and the posterior part of the frontal is the long dermopterotic. The dermopterotic is a subrectangular bone, directed backward, and witha very delicate posterior widening. The posteriormost bone of the cranial roof is the extrascapular. This bone is much smaller than the parietal, subtriangular in shape, and tapering medially. The occipital sensory canal extends through the anterior part of the extrascapulars. Anteriorly, the extrascapular partly overlaps the posterior margins of the parietal and the dermopterotic; posteriorly, it overlaps the anterior margin of the posttemporal; and laterally, it contacts the dorsal part of the opercle.

The circumorbital series is completely preserved in the specimen IGM.6605 (Figure 4). It is represented by the lacrimal, four infraorbitals (one subinfraorbital and two postinfraorbitals), the dermosphenotic, and two supraorbital bones.

The first infraorbital (= lacrimal) is longer than deep, and triangular in lateral view; its posterior margin is slightly more tapered than the anterior border; the bone lies in ventral contact with the anterior third of the maxilla and articulates anteriorly with the antorbital. The first infraorbital is followed by a well-developed subinfraorbital. The subinfraorbital is then followed by one (Figure 4) or two postinfraorbitals (Figure 5): The anterior one, in the posterior corner of the orbit, is divided into a massive expanded ventral part and a vertical ramus and the posterior and dorsal one, narrow and vertically positioned reaching the dermosphenotic dorsally.

The dermosphenotic (Figures 4, 5) is a somewhat rectangular bone that participates in the posterodorsal angle of the orbit, below the frontal and in front of the dermopterotic. It contacts the postorbital posteriorly, and the second postinfraorbital ventrally.

There are two articulated rectangular supraorbitals forming the border of the frontals (Figure 4).

The sclerotic ring is not preserved. However, before the preparation of specimen IGM.6605, a thin and curved ossification, here interpreted as a broken sclerotic ring, was noticed.

The posterior cheek region is completely closed with the postorbital plates and the preopercle (Figures 4, 5). Three postorbital plates are present, lying along the anterior edge of the preopercle; the dorsal postorbital is the larger, the other two respectively decrease in size. The preopercle is a narrow crescent-shaped bone, widest near its center. The preopercular sensory canal extends along the entire length of this bone and some large pores occur along its anterior edge. The dorsal edge of the preopercle contacts the dermopterotic, where the preopercular canal communicates with the supratemporal canal.

As often in fossil halecomorphs, very little ofthe braincase is preserved in any of the known specimens of *Cipactlichthys*. The parasphenoid is preserved in lateral view in the holotype (Figure 4).

The premaxilla consists in two main parts: the anterior, laterally elongated, oral border and a posterior process. The anterior oral process of the premaxilla bears the largest teeth of the jaws, aligned in a single row.

The jaws extend well below the posterior orbital margin, reaching the posterior third of the postorbital plate.

The maxilla is elongated, and increases in depth posteriorly. It has a convexly rounded posterior margin (Figures 4, 5). The dorsal edge of the posterior part of the maxilla is excavated for the tightly fitting supramaxilla. As in the premaxilla, the oral border of the maxilla bears a single row of teeth. These teeth are slightly smaller than those of the premaxilla, becoming smaller posteriorly. The lateral surface of the maxilla and the supramaxilla bear longitudinal ridges of ornamentation. The small supramaxilla articulates with the posterior fourth of the dorsal surface of the maxilla (Figure 4).

The dentary is long and slender, increasing its height in the rear (Figures 4, 5). The symphysis is medially curved. The dentary posteriorly sutures with the angular, while the angular sutures dorsally with the supraangular and posteriorly with the retroarticular. Two articular ossifications respectively articulate with the quadrate and the symplectic (see below).

The outer surface of the dentary is ornamented with longitudinal striations, and bears the pore openings of the mandibular sensory canal that extends along the ventral part of the bone. This sensory canal is continuous with a similar series on the angular and with the preopercular canal.

The palatal complex is not well preserved in either specimen. However, in IGM.6606 the oral face of the ectopterygoid and the endopterygoid is visible (Figure 5). The ectopterygoid is a thin and elongated bone bearing a thin shagreen of tiny teeth on its oral surface. The endopterygoid also has tiny villiform teeth on its oral surface.

**Figure 6:**
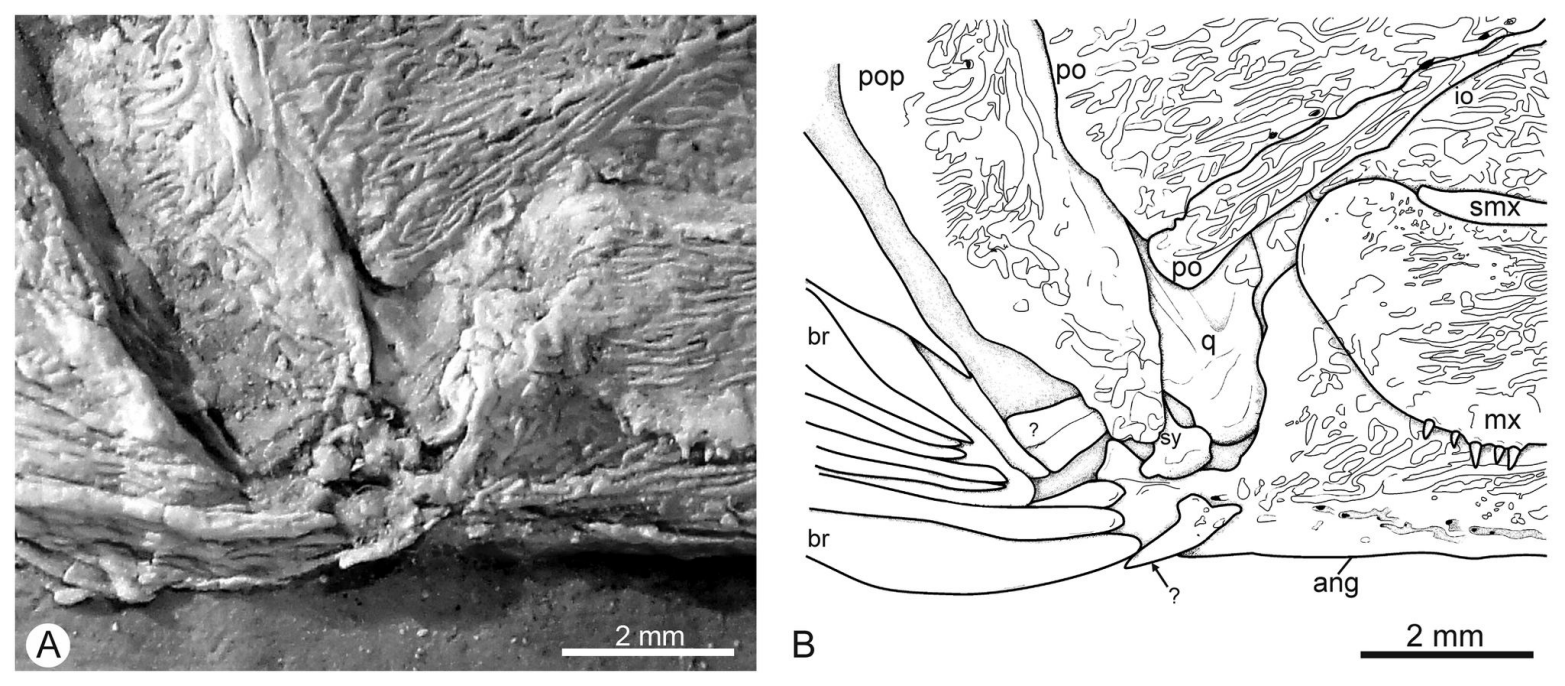
*Cipactlichthys* *scutatus* gen. et sp. nov. Holotype IGM.6605. Detail of the double articulation with the lower jaw. A) photograph; B) anatomical interpretation. Abbreviations: ang, angular; br, branchiostegals; io, infraorbital; mx, maxilla; po, postorbital; pop, preopercle; q, quadrate; sy, sympletic. Scale bar equals 2 mm.

The quadrate is fan-shaped with a well-developed condyle. The symplectic is overlain by the lower part of the preopercle and also has a well developed, anterior-oriented condyle, indicating a double articulation with the jaw (Figure 6).

The opercular series consists of three bones: the opercle, the subopercle, and the interopercle (Figure 4). The opercle is almost twice as high as wide. The anterior margin of the opercle is vertical and straight, and partially covered by the preopercle; posteriorly, the opercle abuts the supracleithrum and the dorsal part of the cleithrum; dorsally, the opercle contacts the ventral margin of the dermopterotic, whereas its ventral border abuts the subopercle.

The subopercle is roughly a four-sided element smaller than the opercle; its anterior margin is deeper than its posterior margin. It articulates with the preopercle anteriorly and contacts the cleithrum posteriorly. The interopercle is small with an elongate triangular outline; it contacts the posterior margin of the preopercle anteriorly.

At least six thin and elongate branchiostegal rays are present on the right side of the skull in specimen IGM.6605 (Figure 4) and located posterior to the maxilla.

A possible short and oval gular plate can be seen in IGM.6606 (Figure 5). This bone is located below the posterior half of the dentary.

The dermal elements of the pectoral girdle include the posttemporal, supracleithrum, cleithrum, and two postcleithra (Figure 4). The clavicle cannot be observed.

The posttemporals are oblique and underlie anteriorly the extrascapulars and their posterior dorsal surface are covered with scales. Their precise shape cannot be observed due to the preservation of our material. These bones accommodate the lateral line canal along their lateral edge.

Underlying the posterolateral corner of the posttemporal is the anterodorsal tip of the supracleithrum. The supracleithrum is thin and underlies the opercle anteriorly, contacts the dorsal end of the cleithrum ventrally, and overlaps the upper part of the postcleithrum posteroventrally.

The dorsal postcleithrum is a somewhat rectangular bone, deeper than long. This bone has nearly the same size as the supracleithrum. The ventral postcleithrum is a much smaller bone. Posteriorly, the postcleithra overlap the first row of scales.

The cleithrum is L-shaped with an anteriorly lower arm that seems to be about two thirds of the upper arm. Anteriorly, it underlies the subopercle; posteriorly, it overlaps the postcleithra.

The scapulocoracoid of *Cipactlichthys* articulates with the median surface of the cleithrum, just behind the ventral part of the dorsal arm of the cleithrum.

The pectoral fin articulates with the round propterygial ossification and the distal radials.

The pectoral fin margins are slightly convex and the shaft of the anteriormost fin ray is lined with fringing fulcra (Figures 4, 5). The pectoral fin has 8-9 principal rays, the first ray being very long, reaching the posterior edge of the pelvic fin (Figure 3). The rays are bifurcated and segmented distally. The leading edge of the anteriormost fin ray is lined with fringing fulcra.

The pelvic girdle of *Cipactlichthys* *scutatus* includes a broadly expanded element, the pelvic bone. Articulating with the posterior end of the pelvic girdle there are about five pelvic fin rays. The pelvic fin rays are branched, and the edge of the fin bears a series of fringing fulcra.

Both the dorsal and the anal ﬁns are triangular and located at the level of the 27^th^ and 28^th^ transverse row of scales, respectively, with a one-to one correspondence to the pterygiophores.

**Figure 7:**
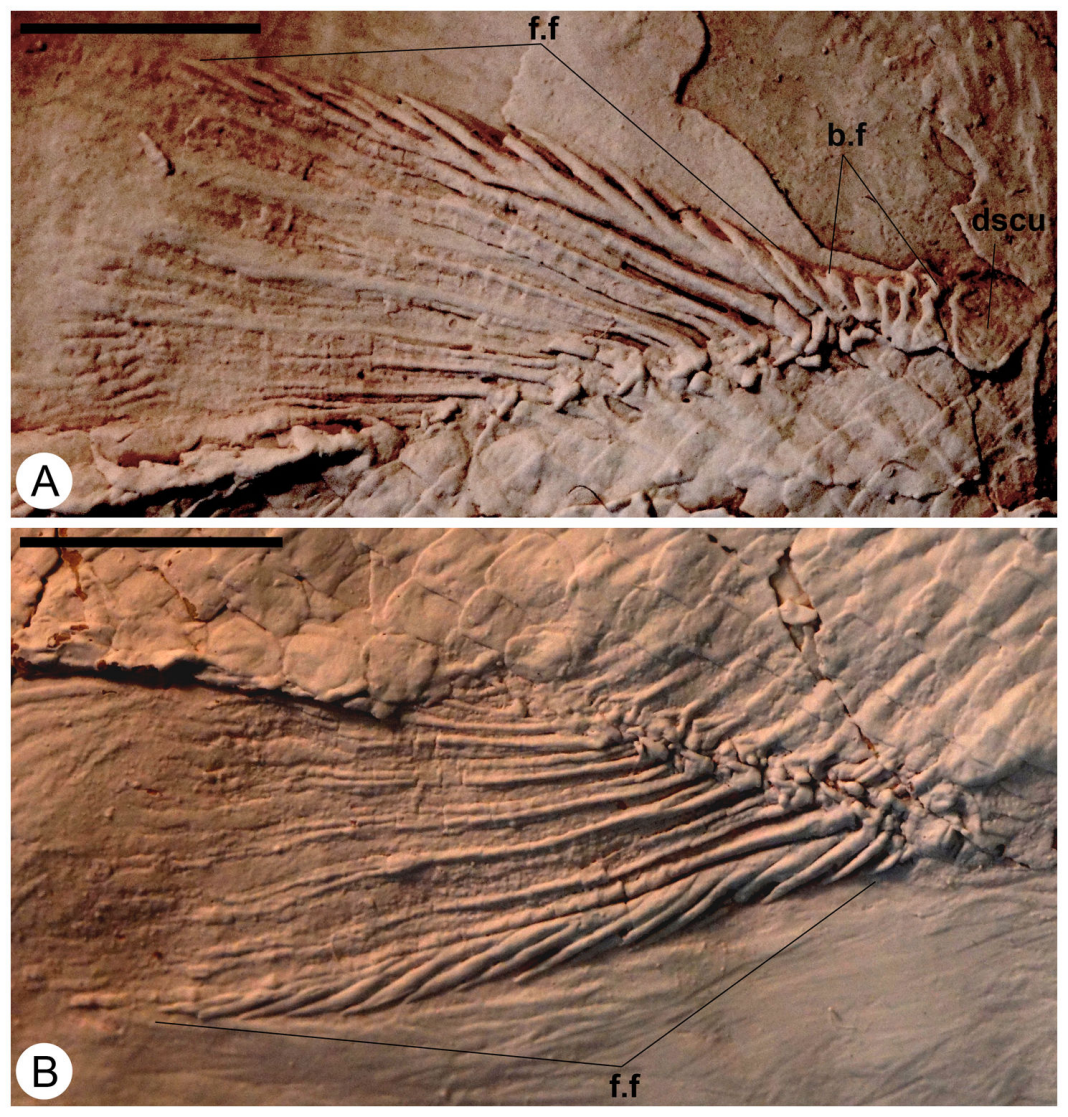
*Cipactlichthys* *scutatus* gen. et sp. nov. Holotype IGM.6605. A) Photograph of dorsal fin. B) Photograph of anal fin. Abbreviations: b.f, basal fulcra; dscu, dorsal scute; f.f, fringing fulcra. Scale bar equals 5 mm.

The dorsal fin (Figure 7A) spans seven or eight scales and bears at least 8 fin rays, the first one unbranched and seven segmented and bifurcated distally. The dorsal fin is preceded by a scute and five basal fulcra; the anterior edge of the fin is lined with fringing fulcra. The anal fin is larger than the dorsal fin (Figures 2, 3), spans about eight scales, and bears at least 8 rays. Like in the dorsal fin, the anal fin is also preceded by a scute, four basal fulcra and the anterior edge of the principal ray is lined with fringing fulcra (Figure 7B).

**Figure 8:**
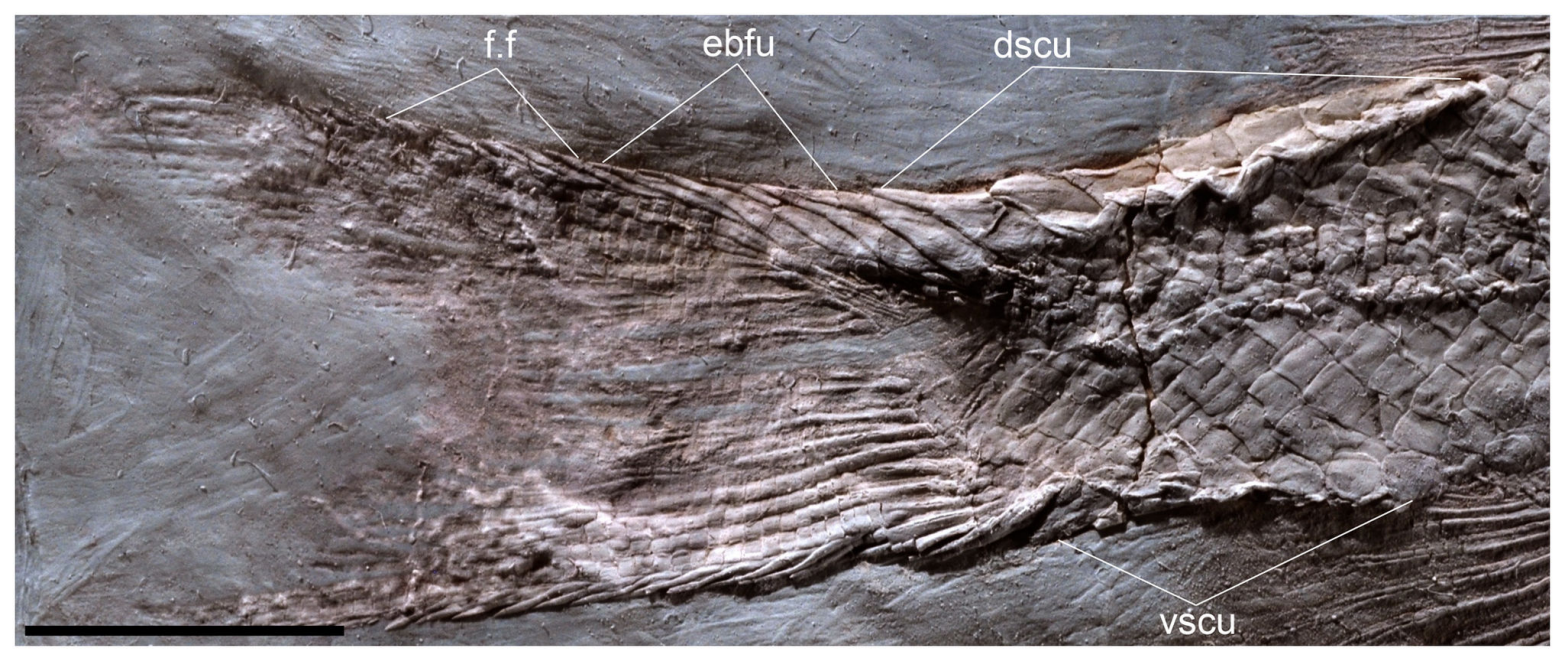
*Cipactlichthys* *scutatus* gen. et sp. nov. Holotype IGM.6605. Photograph of the posterior region of the body. Abbreviations: dscu, dorsal scute; ebfu, epaxial basal fulcra; f.f, fringing fulcra. Scale bar equals 5 mm.

The caudal fin has a forked outline, with both lobes of equal size (Figures 2, 3). The total number of branched caudal fin rays is fourteen. The upper lobe contains 7 rays. Scutes precede the series of epaxial basal fulcra. The series of basal fulcra decrease in width caudally and each fulcrum overlaps the next one. A series of fringing fulcra lies on the dorsal margin of the first principal ray (Figure 8).

There are seven rays in the lower lobe. The ventral caudal scute precedes two (or three) hypaxial basal fulcra. The first two rays (procurrent rays) are short and do not seem to support any fulcra (Figure 8). The next ray corresponds to the lowermost segmented-and-branched principal ray. Fringing fulcra extend along the entire ventral margin of the caudal fin. They decrease progressively posteriorly.

Scutes, basal fulcra, fringing fulcra, and lepidotrichia seem to be completely covered with ganoine.

*Cipactlichthys* has solid ossified smooth-sided centra, deeper than long. Diplospondyly is not observed. The number of preural vertebrae is about 37, with sixteen abdominal centra. The caudal centra involve 14 preural and 7 ural centra.

**Figure 9:**
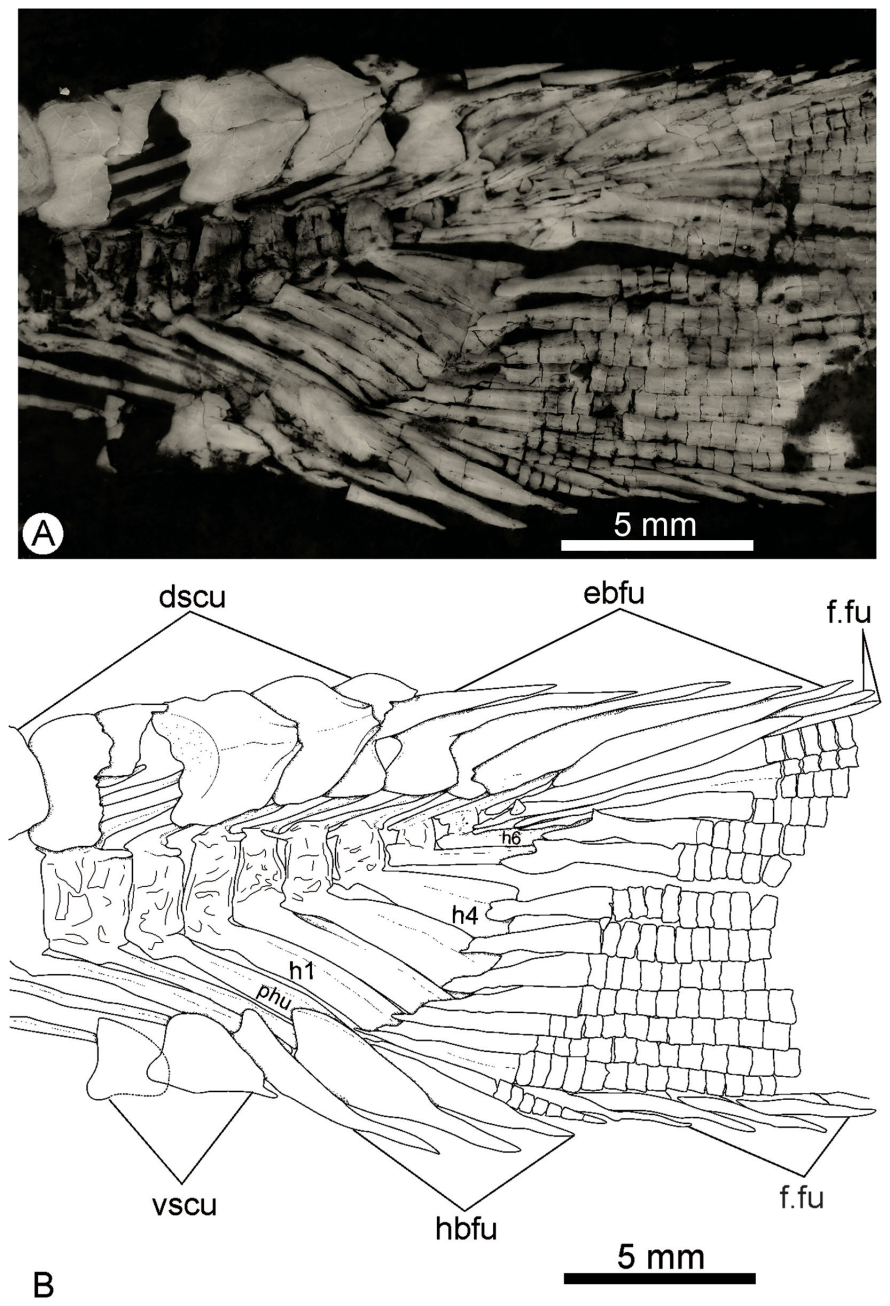
*Cipactlichthys* *scutatus* gen. et sp. nov. IGM.6606. A) Photograph of caudal fin, with detail of endoskeleton. B) anatomical interpretation. Abbreviations: b.f, basal fulcra; dscu, dorsal scute; ebfu, epaxial basal fulcra; f.f, fringing fulcra; H, hypurals; hbfu, hipaxial basal fulcra; PHu, parhypural; vscu, ventral scutes. Scale bar equals 5 mm.

Six or seven hypurals can be observed. They are fused to their centra and each one corresponds to more than one ray (Figure 9). Epurals could not be observed in the specimens; there is one urodermal.

The scales are strongly ornamented, lacking serrations along the posteriormargin. The presence of ganoine is highlighted by the use of ultraviolet (UV) light, because this tissue exhibits a yellowish color when photographed under this light.

There is much variation in the size and shape of the scales. There areabout 38 scales on the lateral line from the supracleithrum to the base of the axial lobe of the caudal ﬁn (Figure 2).

The flank scales are arranged with two rows of deep scales, consisting of lateral line scales, about three to four times higher than wide, and an upper row of scales of the same size. These scales decrease in height posteriorly. Dorsal to the flank scales there are three rows of much smaller scales. Ventral to the flank scales there are four or five rows of small, rectangular scales, only slightly higher than wide.

*Cipactlichthys* presents a series of scutes garnishing the dorsal and ventral midline, between the unpaired fins and the caudal fin (Figures 2, 3). The dorsal scutes form a series of seven pentagonal-shaped elements. These elements present a long median longitudinal crest and a well developed spine (Figures 8, 9). The ventral scutes are hexagonal and are smooth and only slightly larger than the adjacent scales and present a tiny median longitudinal crest. A median scute is placed at the origin of the dorsal and the anal fin.

### Phylogenetics

The data matrix (Table S1) was run using PAUP 4b10 [18] with default options and performed the analysis with ACCTRAN optimization.

*Cipactlichthys* was examined and compared with the skeletons of 12 other actinopterygians. Two taxa, *Polypterus* *senegalus* , representing the polypterid, and *Acipenser* *brevirostrum* representing the acipencerid, were considered as outgroups. The remaining 10 ingroup taxa were all neopterygians. We chose for this analysis *Watsonulus* *eugnathoides* , representing Parasemionotidae and considered as most basal within the halecomorphs [1], and representatives of two halecomorph orders: the Ionoscopiformes, here represented by the ophiopsid *Placidichthysbidorsalis* and the oshuniid *Oshunia* *brevis* ; and the Amiiformes Amia calva and *Callamopleurus* *cylindricus* . We also included the semionotid *Lepidotes piauhyensis*, two lepisosteiforms: *Dentilolepisosteus* *laevis* (Obaichthidae) and *Atractosteus* *tropicus* (Lepisosteidae), as well as the ichthyodectiform *Cladocyclus* *gardneri* and *Elops* *saurus* (Elopidae). All taxa were examined from original material.

**Figure 10:**
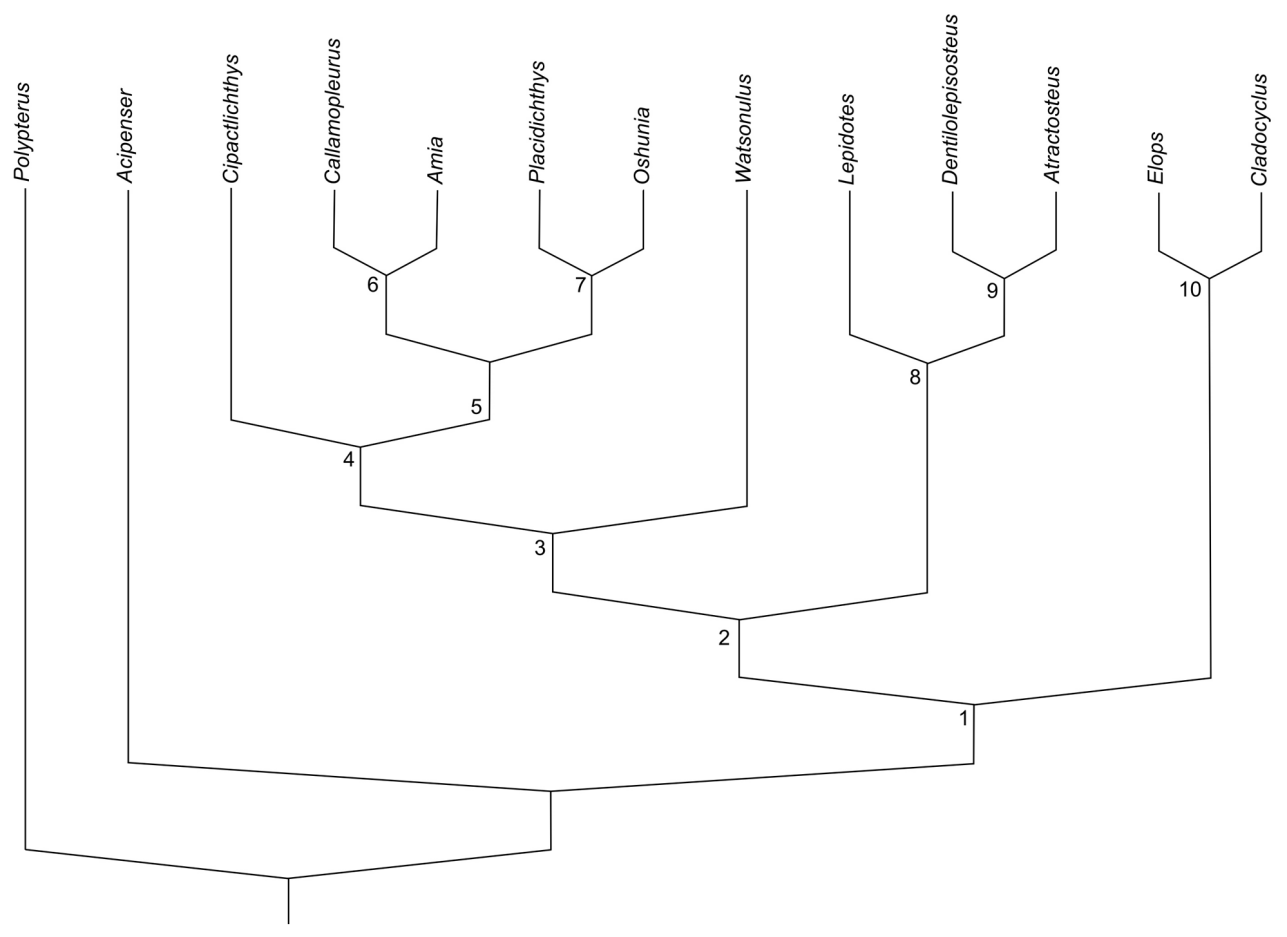
Most parsimonious tree with length of 84 (CI = 0. 7024, HI = 0. 2976, RI = 0. 7768, and RC = 0. 5442). Nodes 1) Neopterygii; 2) Holostei; 3) Halecomorphi; 4) Unnamed component 4, 5) Unnamed component 5, 6) Ionoscopiformes; 7) Amiiformes; 8) Ginglymodi; 9) Lepisosteiformes; 10) Teleosts.

Our parsimony analysis running a heuristic search resulted in one most parsimonious tree, with a length of 82 (CI = 0. 6951, HI = 0. 3049, RI = 0. 7664, and RC = 0. 5327). The topology displays a total of 10 components (Figure 10).

The neopterygians (node 1, bootstrap value equals 100) are confirmed as a monophyletic group supported by three synapomorphies: presence of a symplectic bone (cha. 27, ci= 1); absence of quadratojugal (cha. 29, ci= 1); and the relationship of fin rays to pterygiophore ratios of the dorsal and anal fins of about 1:1 (cha. 33, ci= 1). Node 1 is also supported by eight homoplastic characters (e.g., presence of supraorbital bones (cha. 7, ci= 0.500); complete circumorbital ring (cha. 8, ci= 0.500); presence of an anterior myodome (cha. 10, ci= 0.500); presence of a supramaxilla (cha. 15, ci= 0.500); presence of one supramaxilla (cha. 16, ci= 0.667); presence of an interopercle (cha. 21, ci= 0.500); the absence of clavicles (cha. 24, ci= 0,500); and the presence of a free maxilla, in this case with a reversion in *Atractosteus* (cha. 49, ci= 0,500)). Our analysis regards the Teleostei as the sister group of the Holostei (Ginglymodi + Halecomorphi). However, a discussion of this clade is beyound the scope of the present work.

The Holostei, resolved at node 2, are well supported (bootstrap value equals 95) by thirteen characters: presence of a tube-line canal bearing the anterior arm on the antorbital (cha. 3, ci= 1); absence of pterotic (cha. 11, ci= 1); two vertebrae fused into the occipital condyle (cha. 12, ci= 1); presence of paired vomers (cha. 13, ci= 1); mandibular coronoid process formed by a compound structure, involving more than one bone. (cha. 18, ci= 1); presence of supraangular (cha. 19, ci= 1); presence of anterior and posterior clavicle elements (cha. 25, ci= 1); presence of four hypobranchials (cha. 26, ci= 1); presence of median and paired neural spines in the caudal region (cha. 32, ci= 1); all caudal fin principal rays are branched. (cha. 36, ci= 1); and the presence of canaliculi of Williamson in the bones (cha. 48, ci= 1). Two other homoplastic characters support the holosteans: the posterior extent of median rostral tube simple at the anterior end of snout with no internasal lamella (cha. 2, ci= 0.667); and the presence of fringing fulcra on the caudal fin (cha. 31, ci= 0.500).

Here we do not consider Ginglymodi as this clade has been recently exhaustively discussed and is very well supported in our analysis by numerous synapomorphies [3,20,21] (bootstrap value equals 98).

The clade formed by the halecomorphs is moderately well supported (node 3 - bootstrap value equals 61) by two characters: a symplectic involved in the jaw joint (cha. 28, ci = 1) and a crescent-shaped preopercle (cha. 20, ci = 1).

*Cipactlichthys* is rooted as the sister group of the remaining halecomorphs (node 4 - bootstrap value equals 61). This pattern of relationship is supported by two shared derived characters: rostral bone roughly V-shaped, with lateral horns (cha.1, ci = 1). Clade 5 (boostrap of 65), is formed by the Ionoscopiformes sensu Grande and Bemis, 1998 [1] and the Amiiformes and is supported by one synapomorphy: posterior margin of maxilla presenting a notch (cha. 14, ci = 1), and by two homoplastic characters: dermosphenotic firmly sutured to the skull roof (cha. 6, ci = 0.33); and the presence of amioid scales, not found in the Ophiopsids, here represented by *Placidichthys* where the plesiomorphic state (ganoid scales) is present (cha. 40, ci = 0.50).

## Discussion

Here we consider the relationships of *Cipactlichthys* within Holostei, the Halecomorphi and clade 4.

The hypothesis of relationships described above confirms the monophyly of the holostei, based on thirteen synapomorphies, and thus agreeing with the results of [3]. *Cipactlichthys* presents five of these synapomorphies (the posterior extent of median rostral tube simple at the anterior end of snout with no internasal lamellae (cha. 2), presence of a tube-line canal bearing the anterior arm on the antorbital (cha. 3); mandibular coronoid process formed by a compound structure, involving more than one bone (cha. 18); the presence of fringing fulcra on the caudal fin (cha. 31); and the presence of canaliculi of Williamson in the bones (cha. 48). The first four characters were discussed by [3] and are not discussed here. However, regarding the presence of canaliculi of Williamson in the bones we note that such structures have been considered as typical "holostean" features [22–24]. They occur in ganoid scales of ginglymodins, halecomorphs and in the teleosteomorphs such as pholidophorids [24,25] and aspidorhynchids [25,26]. These structures also occur in the bones of holosteans. Canaliculi of Williamson are not found in palaeoniscoids, in fossil or extant polypterids or in modern teleosts. Among halecomorphs, they are not present in the elasmoid amioid scales.

We corroborate the monophyly of the halecomorphs. However, the level of distribution of some characters differ somewhat from those in literature [1,27] where this clade is supported mainly by three features (e.g., an elongated symplectic forming part of the double articulation with the lower jaw; the presence of a notch in the posterior margin of the maxilla; and the presence of a single supramaxillary bone). A phylogenetic study of halecomorphs is the subject of a work in preparation by Ms. Giselle Machado in the Universidade do Estado do Rio de Janeiro. Accordingly, we will only discuss the characters related to the definition of the halecomorphs and/or important for the relationships within this group.

Our tree shows *Watsonulus* at the basal level of the halecomorphs corroborating this taxon as the sister group of all the other members of the Halecomorphi (node 3). However, in our analysis, node 3 (= Halecomorphi) is supported only by two characters: the symplectic forming part of the double articulation with the lower jaw and a crescent-shaped preopercle.

A symplectic forming part of the double articulation with the lower jaw was first considered as an halecomorph synapomorphy by [2] and was accepted by many subsequent authors [1,3,27–29]. A double jaw articulation, quadrate/symplectic, is also found in the aspidorhynchid *Vinctifer* [30,31]. The presence of a double articulation in *Vinctifer* was interpreted as a reversal of this character state in this taxon (not known in the other genera of the family), and therefore homoplastic with the Halecomorphi.

A crescent-shaped preopercle was considered in our analysis as a synapomorphy for halecomorphs, with a reversal to the state "ovoid shape preopercle" in *Watsonulus* . However, in our interpretation a crescent-shaped preopercle should be regarded as a synapomorphy that supports component 4. The state of this character in *Watsonulus* is used to support the monophyly of parasemionotids [27]. However, the shape of the preopercle differs among the members of this family and a comprehensive systematic study of this group is waiting [27].

The presence of a notch in the posterior margin of the maxilla in *Watsonulus* is questionable because in some of the specimens examined this notch is absent. This notch is also absent in *Cipactlichthys* (Figures 4, 5) and therefore considered in our analysis as a synapomorphy for node 5 (Ionoscopiformes + Amiiformes). A notch in the posterior margin of the maxilla is also found in certain pholidophoriforms and in the basal teleost *Varasichthys* [27].

The presence of a single supramaxilla is considered in our analysis as a synapomorphy of the neopterygians. Halecomorphs possess a single supramaxilla, as well as the teleosteomorph *Aspidorhynchus* and *Belonostomus* [31], and many basal teleosts such as the extant albulid *Albula* *vulpes* .

One character supports clade 4, the node formed by *Cipactlichthys*, (Ionoscopiformes + Amiiformes): an approximately V-shaped rostral bone, with lateral horns.

In *Watsonulus* the rostral bone is a median plate carrying the ethmoidal commissure, a condition similar to that found in polypterids [32], ginglymodins [3] and teleosteomorphs such as aspidorhynchids and pholidophorids [31,33]. The condition in the other halecomorphs (not *Watsonulus* , Parasemionotidae) is roughly V-shaped, with lateral horns containing the commissural canal. The condition in members of the major teleost lineages is very different as an independent rostral bone is absent [27]. For this reason we coded this character as non-applicable. In *Elops* the sensory comissure canal passes from the dermethmoid to a chain of rostral ossicles and in *Cladocyclus* there is a complex “rostrodermethmoid,” in which this comissure canal is not observed.

## Conclusions

This study clearly demonstrates the presence of a new species of neopterygian, *Cipactlichthys* *scutatus* gen. et sp. nov. in the Tlayua Formation, Lower Cretaceous of Mexico.

*Cipactlichthys* is recovered as a member of the Halecomorphi based on its possession of two synapomorphies: the crescent-shaped preopercle and a symplectic involved in the jaw articulation. However, its position within the Halecomorphi needs further investigation, but only after considerable work on other fossil halecomorphs has been undertaken.

Our results support a relationship between *Cipactlichthys* *scutatus* and the ionoscopiforms and amiiforms, based mainly on the rostral bone being approximately V-shaped, and bearing lateral horns. However, *Cipactlichthys* *scutatus* differs from Ionoscopiformes and Amiiformes by the posterior margin of the maxilla being convexly rounded (*contra* a posterior margin with a notch or excavated), by its dermosphenotic loosely attached to the skull roof (*contra* a dermosphenotic firmly sutured to the skull roof), and by the ganoid type of scales, also found in the ophiopsids but different in the oshuniids, ionoscopids, caturids and amiids.

*Cipactlichthys* differs from all other halecomorph taxa in the jaw length, ornamentation of the dermal bones and scales, length of the pectoral fin, a series of hypertrophied scales just posterior to the cleithrum, the arrangement of the flank scales with two rows of deep scales, and a series of dorsal and ventral scutes forming the dorsal and ventral midline, between the dorsal and anal fins and the caudal fin among others.

Finally, the present analysis is far from being completely satisfying as the node supporting the clade Halecomorphi, as well as the clade formed by *Cipactlichthys* (Ionoscopiformes + Amiiformes), are weakly supported. Therefore a systematic revision of of the Halecomorphi, including the basal members including Triassic parasemionotiforms and species such as *Cipactlichthys* *scutatus* and *Brachydegmacaelatum* Dunkle, 1939 [34], from the Early Permian, will be essential for a better understanding of the phylogenetic interelationships within the halecomorphs. However, it provides a preliminary step which needs to be tested using new material and new discussions of the characters deﬁned for the phylogenetic analysis.

## Supporting Information

Table S1 Data matrix for phylogenetic analysis.
(PDF)

Text S1 List of material examined.
(PDF)

Text S2 **List of characters used in the phylogenetic analysis.**
(PDF)
