# Supplementary material for: Cipactlichthys scutatus, gen. nov., sp. nov. a New Halecomorph (Neopterygii, Holostei) from the Lower Cretaceous Tlayua Formation of Mexico
Source: PLoS One. 2013 Sep 4;8(9):e73551. doi: 10.1371/journal.pone.0073551 (PMC3762789; doi:10.1371/journal.pone.0073551)
Supplement: Table S1 — Data matrix for phylogenetic analysis. (PDF) [file pone.0073551.s001.pdf]

Supplementary Table 1. Data matrix.

**Taxa**	**1**	**2**	**3**	**4**	**5**	**6**	**7**	**8**	**9**	**10**	**11**	**12**	**13**	**14**	**15**	**16**	**17**	**18**	**19**	**20**	**21**	**22**	**23**	**24**	**25**	**26**	**27**	**28**	**29**	**30**	**31**	**32**	**33**	**34**	**35**	**36**	**37**	**38**
Polypterus	0	0	-	0	0	?	0	0	0	1	?	1	0	-	0	0	1	0	0	2	1	0	0	0	0	0	0	-	0	?	0	1	0	1	2	-	0	0
Acipenser	-	2	-	-	-	1	0	0	0	1	-	3	0	-	0	-	0	0	0	-	1	1	0	0	0	0	0	-	0	0	0	1	0	0	1	0	0	-
Cipactlichtys	1	1	1	0	0	0	1	1	0	?	?	?	?	0	1	1	1	1	?	1	0	0	?	?	?	?	1	1	2	0	1	?	1	0	?	?	0	?
Watsonulus	0	1	1	0	0	0	1	1	0	0	1	?	1	0/1	1	1	1	1	1	2	0	0	?	0	?	?	1	1	2	0	1	?	1	0	?	?	0	?
Placidichthys	1	1	1	0	0	1	1	1	0	0	1	?	1	1	1	1	1	1	1	1	0	0	?	?	?	?	1	1	2	0	1	?	1	0	?	?	0	?
Oshunia	1	1	1	0	0	1	1	1	0	0	1	?	1	1	1	1	1	1	1	1	0	0	0	1	?	1	1	1	2	0	1	?	1	0	1	1	0	0
Amia	1	0	1	0	0	1	0	-	0	0	1	2	1	1	1	1	1	1	1	1	0	0	0	1	1	1	1	1	2	1	0	3	1	1	1	1	1	0
Callamopleurus	1	1	1	0	0	0	1	1	0	0	1	?	1	1	1	1	1	1	1	1	0	0	0	1	?	1	1	1	2	1	0	3	1	1	1	1	1	0
Lepidotes	0	1	1	1	1	0	1	1	0	?	?	-	?	0	1	1	0	1	1	?	0	1	1	1	-	1	1	0	1	0	1	3	1	0	1	1	?	-
Dentilolepisosteus	0	1	1	1	1	0	1	1	0	1	1	2	1	0	1	1	0	1	1	0	0	1	1	1	?	?	?	?	1	0	1	3	1	1	0	1	1	-
Atractosteus	0	1	1	1	1	0	1	1	0	1	1	2	1	0	0	0	1	1	1	0	1	1	1	1	1	1	1	0	1	0	1	3	1	1	0	1	1	0
Elops	-	2	0	0	0	0	1	0	1	0	0	0	0	0	1	2	1	0	0	0	0	0	0	1	0	0	1	0	2	1	0	1	1	0	1	0	0	1
Cladocyclus	-	2	0	1	0	0	1	1	1	0	0	?	0	0	1	2	1	0	0	0	0	1	0	1	0	?	1	0	2	1	0	1	1	0	1	0	0	1

**Taxa**	**39**	**40**	**41**	**42**	**43**	**44**	**45**	**46**	**47**	**48**	**49**
Polypterus	0	0	0	1	0	0	0	0	0	0	0
Acipenser	0	1	0	-	-	-	0	-	0	0	-
Cipactlichtys	0	0	0	0	0	0	0	0	0	1	1
Watsonulus	0	0	0	0	0	0	0	0	0	1	1
Placidichthys	0	0	1	1	0	0	0	0	0	1	1
Oshunia	0	1	1	1	0	0	0	0	0	1	1
Amia	0	1	0	0	0	0	0	0	0	1	1
Callamopleurus	0	1	0	0	0	0	0	0	0	1	1
Lepidotes	0	0	0	0	0	0	0	0	0	1	1
Dentilolepisosteus	0	0	0	0	1	1	1	1	1	1	1
Atractosteus	0	0	0	0	1	1	1	1	1	1	0
Elops	1	2	0	0	0	0	0	0	0	0	1
Cladocyclus	1	2	0	0	0	0	0	0	0	0	1
