# Supplementary material for: Cipactlichthys scutatus, gen. nov., sp. nov. a New Halecomorph (Neopterygii, Holostei) from the Lower Cretaceous Tlayua Formation of Mexico
Source: PLoS One. 2013 Sep 4;8(9):e73551. doi: 10.1371/journal.pone.0073551 (PMC3762789; doi:10.1371/journal.pone.0073551)
Supplement: Text S1 — List of material examined. (PDF) [file pone.0073551.s002.pdf]

Supplementary Text 1. List of material examined.

Institutional abbreviations. DGM, Divisão de Geologia e Mineralogia, Departamento Nacional de Produção Mineral, Rio de Janeiro, Brazil; IGM, Colección Nacional de Paleontología, Instituto de Geologia, Universidad Nacional Autónoma de México, D.F., Mexico; MNHN, Muséum National d'Histoire Naturelle, Paris, France; UERJ, Universidade do Estado do Rio de Janeiro, Rio de Janeiro, Brazil.

Polypterus senegalus Cuvier, 1829 [1]: UERJ cd3, 4, 5, 10.

Asipenser brevirostrum Lesueur, 1818 [2]: UERJ cd8, 11,12.

Watsonulus eugnathoides (Piveteau, 1935) [3]: MAE-83(a,b), MAE-108 (a,b), MNHN MAE-583 (a,b).

Cipactlichtys scutatus: IGM 6605 (Holotype), 6606.

Placidichthys bidorsalis Brito, 2000 [4]: UERJ PMB-300 (a, b), 301, 314.

Oshunia brevis Wenz and Kellner, 1986 [5]: UERJ PMB-394, 395.

Amia calva Linnaeus, 1766 [6]: UERJ cd1, 2, 6, 7, 9.

Calamopleurus cylindricus Agassiz, 1841[7]: UERJ PMB 23, 44, 45, 62.

Lepidotes piauhiensis Roxo and Lofgren, 1936 [8]: DGM 297-P (Holotype), 292-P, UERJ Pz-408, 409, 410.

Dentilolepisosteus laevis (Wenz and Brito, 1992) [9]: DGM-155P (Holotype), UERJ PMB 233, 234.

Atractosteus tropicus Gill, 1863 [10]: UERJ PNT 34, 36, 37.

Elops saurus Linnaeus, 1766 [6]: UERJ PNT 200, 201, 202.

Cladocyclus gardneri Agassiz, 1841 [7]: UERJ-PMB 510, 522, 525.

REFERENCES

1. Cuvier G (1829) Le Règne Animal, distribué d'après son organisation, pour servir de base à l'histoire naturelle des animaux et d'introduction à l'anatomie comparée. Vol 2: i-xv + 1-406.

2- Lesueur CA (1818) Description of several species of chondropterigious fishes of North America. Transactions of the American Philosophical Society, n.s., 1: 383–394.

3- Piveteau J (1935) Paléontologie de Madagascar. XXI. Les Poissons du Trias infkrieur. Contribution à l'étude des actinoptérygiens. Annales de Paléontologie 23: 8 1-180.

4- Brito PM (2000) A new halecomorph with two dorsal fins, *Placidichthys bidorsalis* n. g., n. sp. (Actinopterygii: Halecomorphi) from the Lower Cretaceous of the Araripe Basin, northeast Brazil. Comptes Rendus de l'Académie des Sciences, Paléontologie 331: 749–754.

5- Wenz S, Kellner AW (1986) Découverte du premier Ionoscopidae (Pisces: Halecomorphi) sud- américain, *Oshunia brevis* n. g., n. sp., dans le Crétacé inférieur de la Chapada do Araripe (nord-est du Brésil). Bulletin de Museum d'Histoire Naturelle de Paris 8 (1): 77–88.

6- Linnaeus C (1766) Systema naturae per regnatria naturae, secundum classes, ordines, genera, species, cum characteribus, differentiis, synonymis, locis. Tomus I: 1 – 532 ; Editio duodecima, reformata. Holmiae (Laurentii Salvii).

7- Agassiz L (1841) On the fossil fishes found by Mr. Gardner in the Province of Ceará, in the North of Brazil. Edinburgh New Philosophical Journal 30, 82–84.

8- Roxo, M., and A. Löfgren (1936) *Lepidotus piauhyensis*, sp. nov. Notas Preliminares e Estudos, DNPM 1:7–12

9- Wenz S, Brito PM (1992) Primière découverte de Lepisosteidae (Pisces, Actinopterygii) dans le Crétacé inférieur de la Chapada do Araripe (N-E, du Brésil). Conséquences sur La phylogénie des Ginglymodi. Comptes Rendus de l'Académie des Sciences, Paléontologie 314(II): 1519-1525.

10- Gill T (1863) Descriptive enumeration of a collection of fishes from the western coast of Central America, presented to the Smithsonian Institution by Capt. John M. Dow. Proceedings of the Academy of Natural Sciences 1863: 162-180.
