# Supplementary material for: Cipactlichthys scutatus, gen. nov., sp. nov. a New Halecomorph (Neopterygii, Holostei) from the Lower Cretaceous Tlayua Formation of Mexico
Source: PLoS One. 2013 Sep 4;8(9):e73551. doi: 10.1371/journal.pone.0073551 (PMC3762789; doi:10.1371/journal.pone.0073551)
Supplement: Text S2 — List of characters used in the phylogenetic analysis. (PDF) [file pone.0073551.s003.pdf]

Supplementary Text 2. List of characters.

1- Shape of rostral bone: 0, plate-like or short tube-like, with lateral horns; 1 roughly V-shaped, with lateral horns. (Grande and Bemis 1998 [1]: character 57).

2- Posterior extent of median rostral: 0, with lamellar bone component separating the nasals, at least anteriorly; 1, simple tube at the anterior end of snoot with no internasal lamella; 2, no autogenous median rostral. (Grande 2010 [3]: character 5).

3- Tube-line canal bearing anterior arm on antorbital: 0, absent; 1, present. (Grande 2010 [3]: character12).

4- Number of lacrimals: 0, only a single lacrimal; 1, a series of 3 or more lacrimals. (Grande 2010 [3]: character 21).

5- Position of anterior lacrimal: 0, part of orbital ring; 1, well anterior to orbital ring. (Grande 2010 [3]: character 20).

6- Dermosphenotic attachment to skull roof: 0, loosely attached on the skull roof or hinged to the side of the skull; 1, firmly sutured. (Grande and Bemis, 1998 [1]: character 56).

7- Supraorbital bones: 0, absent; 1, present. (Grande 2010 [3]: character 14).

8- Circumorbital ring: 0, incomplete; 1, complete (Grande 2010 [3]: character 15).

9- Supraoccipital bone: 0, absent; 1, present. (Grande 2010 [3]: character 28).

10- Anterior myodome: 0, present; 1, absence. (Grande 2010 [3]: character 31).

11- Pterotic: 0, present; 1, absent. (Grande 2010 [3]: character 29).

12- Vertebrae fused into occipital condyle: 0, no vertebral centra fused into centra; 1, one

vertebral centrum fused into condyle during early ontogeny; 2, two vertebrae (after Grande 2010 [3]: character 27).

13- Vomer in adults: 0, median; 1, paired. (Grande 2010 [3]: character 37).

14- Shape of posterior margin of maxilla: 0, convexly rounded or straight; 1, excavated. (Grande and Bemis, 1998 [1]: character 62).

15- Supramaxilla: 0, absent; 1, present. (Grande 2010 [3]: character 47).

16- Number of supramaxillae: 0, none; ,1, one; 2, two . (Grande and Bemis, 1998 [1]: character 69).

17- Mandibular length as a percentage of head length: 0, less than 43%; 1, more than 44%. (Grande 2010 [3]: character 50).

18- Type of mandibular coronoid process: 0, absent or of a single bone; 1, compound structure involving more than one bone. (Grande 2010 [3]: character 51).

19- Supraangular: 0, absent; 1, present. (Grande 2010[3]: character 53).

20- Shape of preopercle: 0, L-shaped; 1, crescent shaped, long and narrow; 2, ovoid. (after Grande and Bemis, 1998 [1]: character 20).

21- Interopercle: 0, present; 1, absent. (Grande 2010 [3]: character 19).

22- Gular: 0, present; 1, absent. (Grande 2010 [3]: character 75).

23- Medial wing on cleithrum: 0 absent; 1 present. (Grande 2010 [3]: character 94.

24- Clavicles: 0, well developed; 1, absent (Grande 2010 [3]: character 95).

25- Anterior and posterior “clavicle elements”: 0, absent; 1, present. (Grande 2010 [3]: character 96).

26- Number of hypobranchials: 0, 3 hypobranchials; 1, 4 hypobranchials. (Grande 2010 [3]: character 99).

27- Symplectic: 0, absent; 1, present. (Grande 2010 [3]: character 67).

28- Symplectic involved in jaw joint: 0, does not articulate with lower jaw; 1, distal end articulates articulates with articular bone of lower jaw. (Grande and Bemis, 1998 [1]: character 61).

29- Quadratojugal: 0, plate-like; 1, splint-like structure with anterior limb of preopercle; 2, absent. (Grande 2010 [3]: character 70).

30- Presence/absence of fringing fulcra on median fins: 0, present; 1, absent. (Grande and Bemis, 1998 [1]: character 45).

31- Fulcra on caudal fin primarly of fringing type: 0, absent; 1, present. (Grande 2010 [3]: character 90).

32- Neural spine type in caudal region: 0, absent; 1, median; 2, paired; 3, median and paired. (Grande 2010 [3]: character 85).

33- Fin Ray to pterygiophore ratios of dorsal and anal fins: 0, 2:1 or greater; 1, about 1: 1. (Grande 2010 [3]: character 89).

34- Shape of posterior margin of caudal: 0, forked; 1, convexly rounded. (Grande and Bemis, 1998 [1]: character 23).

35- Number of principal caudal fin rays in adult: 0, 11 to 13; 1, usually more than 12; 2, usually less than 12. (Grande 2010 [3]: character 88).

36- Caudal fin Ray branching: 0, two or more unbranched principal rays in caudal fin; 1, all principal rays are branched. (Grande 2010 [3]: character 87).

37- One to one arrangement of hypurals and caudal fin rays: 0, last few hypurals each articulate with the bases of several caudal fin rays; 1, each hypural normaly bears a single caudal ray. (Grande and Bemis, 1998 [1]: character 46).

38- Number of Ural centra: 0, count of 4 or more; 1, normally two or less. (Grande 2010 [3]: character 83).

39- Uroneural arches modified as elongate uroneurals: 0, no; 1, Yes. (Grande 2010 [3]: character 86).

40- Type of scale: 0, ganoid; 1, amioid; 2, cicloid. (Brito 1997 [30]: character 38).

41- Innerorbital flange of dermosphenotic: 0, smooth with sensory canal; 1, bearing sensory canal tube. (Grande and Bemis 1998 [1]: character 63).

42- Presence/ absence of lateral line canal in maxilla (0) absent; (1) present. (Grande and Bemis 1998 [1]: character 60).

43- Anterior extent of preopercle: 0, not reaching below anterior part of orbit; 1, reaching below anterior part of orbit. (Grande 2010 [3]: character 17).

44- Supraorbital canal incorpored into premaxilla: 0, no; 1, yes. (Grande 2010 [3]: character 24).

45- Position of lower jaw/quadrate articulation: 0, under or posterior to orbit; 1, in advance of orbit. (Grande 2010 [3]: character 48).

46- Quadrate/Metapterygoid contact: 0, present; 1 absent. (Grande 2010 [3]: character 60).

47- Opisthocoelus vertebral centra: 0, absent; 1, present. (Grande 2010 [3]: character 82)

48- Canaliculi of Williamson on the bones: 0, absent; 1, present.

49- Maxilla: 0- fixed, 1- free. (Brito 1997 [30]: character 18).
